# Prediction of infarction development after endovascular stroke therapy with dual-energy computed tomography

**DOI:** 10.1007/s00330-016-4412-5

**Published:** 2016-06-02

**Authors:** Tanja Djurdjevic, Rafael Rehwald, Michael Knoflach, Benjamin Matosevic, Stefan Kiechl, Elke Ruth Gizewski, Bernhard Glodny, Astrid Ellen Grams

**Affiliations:** 1 Department of Neuroradiology, Medical University of Innsbruck, Anichstraße 35, A-6020 Innsbruck, Austria; 2 Department of Radiology, Medical University of Innsbruck, Innsbruck, Austria; 3 Department of Neurology, Medical University of Innsbruck, Innsbruck, Austria

**Keywords:** Dual-energy computed tomography, Blood-brain barrier disruption, Endovascular stroke therapy, Intracranial haemorrhage, Cerebral infarction

## Abstract

**Objectives:**

After intraarterial recanalisation (IAR), the haemorrhage and the blood-brain barrier (BBB) disruption can be distinguished using dual-energy computed tomography (DECT). The aim of the present study was to investigate whether future infarction development can be predicted from DECT.

**Methods:**

DECT scans of 20 patients showing 45 BBB disrupted areas after IAR were assessed and compared with follow-up examinations. Receiver operator characteristic (ROC) analyses using densities from the iodine map (IM) and virtual non-contrast (VNC) were performed.

**Results:**

Future infarction areas are denser than future non-infarction areas on IM series (23.44 ± 24.86 vs. 5.77 ± 2.77; *p* < 0.0001) and more hypodense on VNC series (29.71 ± 3.33 vs. 35.33 ± 3.50; *p* < 0.0001). ROC analyses for the IM series showed an area under the curve (AUC) of 0.99 (cut-off: <9.97 HU; *p* < 0.05; sensitivity 91.18 %; specificity 100.00 %; accuracy 0.93) for the prediction of future infarctions. The AUC for the prediction of haemorrhagic infarctions was 0.78 (cut-off >17.13 HU; *p* < 0.05; sensitivity 90.00 %; specificity 62.86 %; accuracy 0.69). The VNC series allowed prediction of infarction volume.

**Conclusions:**

Future infarction development after IAR can be reliably predicted with the IM series. The prediction of haemorrhages and of infarction size is less reliable.

***Key Points*:**

• *The IM series (DECT) can predict future infarction development after IAR.*

• *Later haemorrhages can be predicted using the IM and the BW series.*

• *The volume of definable hypodense areas in VNC correlates with infarction volume.*

## Introduction

Dual-energy computed tomography (DECT) is a novel method based on the application of radiation with two different energies that allows different materials with similar densities to be distinguished on conventional computed tomography (CT) [1–4]. For example, haemorrhage can be distinguished from blood-brain barrier (BBB) disruptions or from a mixture of haemorrhage and BBB disruption in different situations [4–11]. Immediately after intraarterial recanalisation (IAR) it is extremely important to detect and distinguish reliably between intracranial haemorrhage and BBB disruptions. This is in order to begin prevention safely of a new ischemic event in patients with no haemorrhage [12] with antiplatelet therapy [13], which is too dangerous in patients with haemorrhage. However, in most cases, hyperdensities immediately after EST represent non-haemorrhagic BBB disruptions [9, 10]; an ischemic infarction may or may not be present, but cannot be diagnosed under these conditions. Haemorrhages may or may not occur in these infarctions in the future, which also cannot be predicted. The larger an infarction is, the more likely it is that there will be haemorrhages later [14]. It is, therefore, also important to detect or predict an infarction under these circumstances and estimate it’s size as soon as possible.

Newer approaches consist of combining rapid reperfusion using IAR with vasculoprotective and neuroprotective treatment in order to improve the outcome even more [15]. Reperfusion and stabilization of the neurovascular unit and BBB must be integrated to prevent adverse reperfusion damage [16]. This combination was recently tested in the URICO-ICTUS trial [17] with promising results. However, despite all efforts to provide the best possible acute treatment, infarctions or haemorrhagic infarctions may still develop later. The first group benefits from secondary prevention of a new stroke [13], while in the latter group, no prostaglandin H2 synthase inhibitors such as aspirin or the like must be administered.

The aim of the present study was to investigate whether DECT, performed immediately after IAR, can be used to predict the future tissue development of non-haemorrhagic BBB disrupted areas.

## Materials and methods

This retrospective study was approved by the local ethical review board (AN2014-0158-337/4.6). Twenty patients (nine women and 11 men; with a mean age of 64.45 ± 14.99 years, range: 24 to 89 years) with a major stroke and the need for endovascular therapy were included. All patients received a DECT scan within 1 h after endovascular stroke therapy, and all patients displayed a BBB disruption in one or more areas (n = 45). No haemorrhagic transformation was present in these areas. During the study period of 2 years, 14 patients who received DECT after EST were excluded due to apparent haemorrhage.

### BBB disrupted areas

The BBB disrupted areas were the putamen in 13 (28.9 %) cases, caudate nucleus in eight (17.8 %), insular cortex in eight (17.8 %), thalamus in seven (15.6 %), a peripheral part of the parietal or frontal lobe in three (6.7 %), part of the occipital lobe in three (6.7 %), part of the cerebellum in two (4.4 %), and part of the pons in one (2.2 %). All 45 regions were discontiguous. To be counted separately as a region with a BBB rupture, the region has to be clearly separated from the others by tissue with no BBB rupture.

### Dual-energy computed tomography

The DECT scans were performed using a SOMATOM Definition Flash CT scanner (Siemens Healthcare GmbH, Erlangen, Germany) with tube voltages of 100 and 140 kVp and a fixed current of 324 mA for both tubes. The dosage was 2.3 ± 0.1 mSv. This was a standard DECT protocol recommended by the vendor. The datasets were reconstructed with 1 mm slice thickness, 0.8 mm increment, H40f medium kernel, base orbit window, and a 200-mm field of view. A 50 % to 50 % weighted standard “brain window” (BW) series was reconstructed (slice thickness: 4 mm, increment: 4 mm, kernel: H30f medium smooth, field of view: 200 mm). Corresponding iodine map (IM) series and virtual non-contrast (VNC) series were reconstructed using the post-processing software “Brain Hemorrhage” (syngo CT Workplace 2012B, Siemens Healthcare GmbH, Erlangen, Germany). A H30f medium smooth kernel was also used for this.

### Image evaluation

Non-haemorrhagic BBB disruption was defined as BW and IM hyperdensity accompanied by VNC hypo- or isodensity, according to Tijssen et al. [10]. These areas were estimated by consensus of two raters who also later performed the density measurements and the volumetries of the areas involved.

**Fig. 1:**
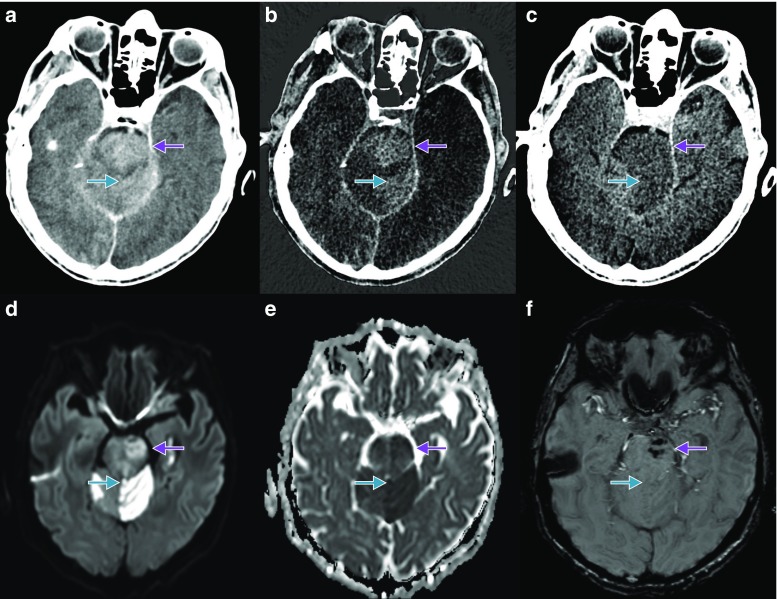
**a**–**f** Example of a patient with a left cerebellar haemispheric infarction (*blue arrow*) and a left pontine infarction (*violet arrow*), which became haemorrhagic 2 days after the IAR. The infarctions are hyperdense on BW series (Figure 1a), hyperdense on IM series (Figure 1b), hypodense on VNC series (Figure 1c), hyperintense on diffusion weighted imaging (DWI B1000, Figure 1d), hypointense on the apparent diffusion coefficient map (ADC map, Figure 1e), hypointense on susceptibility weighted imaging (pontine, haemorrhage), or isointense (cerebellar, no haemorrhage; Figure 1f) in the follow-up imaging

The density measurements in Hounsfield units (HU) on the BW, the IM, and the VNC series (Fig. 1) were performed by two neuroradiologists (TD and AEG) independently, using circular ROI measurements in each of the one to four affected areas per patient. The ROI size was adapted to the size of the area – maximum 1 cm^2^ – on a PACS workstation (IMPAX EE R20 XIV, Agfa, Mortsel, Belgium). Both raters were blinded with respect to the follow-up imaging and to each other. First, three ROIs were placed in the BBB disrupted area in the IM series to represent different parts of the area especially in large areas without touching its edges. Then the three IM ROIs were automatically copied on the BM and VNC series. All measurements were repeated by the two raters several weeks later.

Only in cases in which an infarction was detected in the follow-up images, but the abnormalities in the region were ill defined in the images made immediately after the intervention, were the ROIs placed retrospectively in the later infarction with knowledge of the follow-up images (n = 4). Then the follow-up imaging was analyzed by the same raters. Differences were decided by consensus and a third rater (BG).

The later fate of the 45 areas was investigated on follow-up MRI (n = 10; including susceptibility weighed imaging series; SWI; MAGNETOM Skyra, Siemens Healthcare GmbH, Erlangen, Germany) or CT scans (n = 10) during the first week after the event. Follow-up imaging was performed between 24 h and 7 days after the treatment. The areas were allocated to the three groups: “no infarction”, “ischemic infarction”, and “haemorrhagic infarction”. “No infarction” was defined as normal brain tissue in every sequence on the follow-up scans, “ischemic infarction” as detectable brain cell death [18], and “haemorrhagic infarction” according to the ECASS definition [19].

In a last step, the volumes of the BBB disrupted or conspicuous areas and the infarctions in the follow-up controls were determined by each rater in each series using an AW workstation version 5 [AW VolumeShare 5 (AW4.6), General Electric Company, Fairfield, CT, USA]. After a few days, these volumetries were repeated.

### Clinical and procedure related parameters

The modified Rankin and National Institute of Health Stroke scores, immediately after admission and 90 days after the event, were determined and noted. The time to IAR onset, duration of IAR procedure, etc., are presented in Table 1. The following devices were used: 15 times Solitaire (Solitaire 2, ev3 Neurovascular, Irvine, CA, USA), six times ERIC (ERIC 3, MicroVention, Inc., Tustin, CA, USA), five times ReVive (ReVive SE, Codman, Johnson & Johnson Corp., New Brunswick, NJ, USA), twice pRESET (pRESET, Phenox GmbH, Bochum, Germany), and once a Trevo (Trevo XP, Concentric Medical, Mountain View, CA, USA).

**Table 1:** Procedure related parameters

Variable	
Time to lysis (min)	91.89 ± 54.17
Time to recanalisation (min)	88.26 ± 55.58
Time to IAR (min)	163.94 ± 52.17
Duration of IAR (min)	85.85 ± 52.54
Device passes per procedure	2.68 ± 1.86
Diameter of BBBD area (mm)	17.99 ± 7.91
Contrast agent (mL)	202.63 ± 84.12
NHISS admission	19.00 ± 7.31
mRS admission	4.80 ± 0.40
Procedures used	
iv-Thrombolysis (yes/no)	11/9
Stent	7
Stent, ia-Thrombolysis	12
Stent, Balloon, ia-Thrombolysis	1
Device combinations used	
Eric	2
Eric, Revive, Preset	1
Solitaire	11
Solitaire, Eric	2
Solitaire, Revive	1
Solitaire, Revive, Preset	1
Revive, Eric	1
Revive, Trevo	1
TICI pre-interventional	
0	15
1	4
2a	1
TICI post-interventional	
2a	3
2b	7
3	10

Abbreviations: *BBBD,* Blood brain barrier disruption; *ia,* Intraarterial; *IAR,* Intraarterial Recanalisation; *iv,* Intravenous; *mL*, millilitres; *mRS*, modified Rankin Scale; *NHISS*, National Institutes of Health Stroke Scale; *TICI*, Thrombolysis in cerebral infarction scale

### Statistical analysis

Descriptive analyses were performed using the Excel software (Microsoft Office 2013, Microsoft Corp., Redmond, WA, USA); the Prism 6 software (GraphPad Prism 6, GraphPad Software, Inc., La Jolla, CA, USA) was used for further statistical analyses and presentation. Intra- and inter-rater reliabilities were calculated using the intraclass correlation coefficient [20] (SPSS 23, IBM Corp., Armonk, NY, USA). A kappa coefficient (SPSS) was used to estimate the intra-observer reliability of the categorical variable “infarction/no infarction”. As the data were not entirely normally distributed, the non-parametric Mann-Whitney test for the comparison of two groups, and the Kruskal-Wallis test together with Dunn’s post hoc test for the comparison of three or more groups were used. The Spearman method was used to test for correlations between two groups. The effectiveness of the different reconstruction algorithms was assessed using receiver operating characteristic (ROC) analyses. Cut-off points for decision-making using the BW, IM, and VNC series were determined according to Youden’s method [21]. True positive (TP), true negative (TN), false positive (FP), and false negative (FN) results, sensitivity, specificity, positive predictive value, negative predictive value, accuracy, and AUC were determined. Accuracy was defined as follows:

$$ Accuracy=\frac{TP+TN}{TP+FP+TN+FN} $$

The significance of the deviation of AUC from the others was determined using the method of Venkatraman, or a bootstrapped method, as appropriate in the pROC package in R (R version 3.2.4) [22, 23]. A *p* < 0.05 was regarded to be significant.

## Results

All BBB disrupted areas were within the regions treated with IAR. Pre-interventional hypodense areas consistent with early signs of infarctions were found retrospectively in the CT in two regions. Both of these regions were in infarction areas that were later very extensive, but not affected by haemorrhagic transformation.

Of the 45 investigated areas, 11 (22.2 %) were unsuspicious on follow-up scans, 24 (55.5 %) became ischemic infarctions, and 10 (22.2 %) ischemic infarctions with haemorrhagic transformation. The inter-rater reliability of the estimate with respect to the presence of haemorrhage was perfect (kappa = 1), that with respect to the presence of an infarction in the follow-up scans was very good (kappa = 0.886). Detailed information about the patients and the procedures is presented in Table 1. The intra- and inter-rater reliability are presented in Table 2, the density measurement results and the results of the volumetries in Table 3.

**Table 2:** Intra- and inter-rater reliability

	Intra-rater reliability (ICC) - Density			
	Rater 1	*p*	Rater 2	*p*
BW	0.858	<0.001	0.984	<0.001
IM	0.881	<0.001	0.972	<0.001
VNC	0.795	<0.001	0.604	<0.001
	Intra-rater reliability (ICC) - Volume			
	Rater 1	*p*	Rater 2	*p*
BW	0.944	<0.001	0.933	<0.001
IM	0.947	<0.001	0.954	<0.001
VNC	0.371	0.005	0.720	<0.001
FUI	0.960	<0.001	0.998	<0.001
	Inter-rater reliability (ICC)			
	Density R1/R2	*p*	Volume R1/R2	*p*
BW	0.993	<0.001	0.880	<0.001
IM	0.990	<0.001	0.443	0.028
VNC	0.871	<0.001	0.151	0.297
FUI	-	-	0.985	<0.001

Abbreviations: *BW,* weighted brain window series; *FUI*, Follow-up imaging; *ICC*, Intra-class correlation coefficient; *IM,* Iodine map series; *R1,* Reader 1; *R2,* Reader 2; *VNC,* Virtual non contrast series

**Table 3:** Measured densities and volumes

	Density and volume measurements						
	Density				Volume		
	Rater 1				Rater 1		
	ROI 1_R1_	ROI 2_R1_	ROI 3_R1_	Mean 1-3_R1_	Volume 1_R1_	Volume 2_R1_	Mean 1-2_R1_
BW	53.31 ± 25.76	52.57 ± 15.53	55.15 ± 31.37	53.68 ± 23.87	2.86 ± 2.29	2.90 ± 2.60	2.88 ± 2.42
IM	18.26 ± 16.37	19.40 ± 19.68	20.69 ± 29.36	19.45 ± 21.58	2.15 ± 1.87	2.02 ± 1.73	2.09 ± 1.78
VNC	31.57 ± 4.76	30.26 ± 4.75	30.72 ± 5.05	30.85 ± 4.53	1.58 ± 3.24	0.99 ± 1.67	1.29 ± 2.14
FUI	-	-	-	-	50.73 ± 84.08	64.37 ± 101.38	57.55 ± 92.42
	Rater 2				Rater 2		
	ROI 1_R2_	ROI 2_R2_	ROI 3_R2_	Mean 1-3_R2_	Volume 1_R2_	Volume 2_R2_	Mean 1-2_R2_
BW	54.06 ± 24.81	53.73 ± 26.90	55.12 ± 28-84	54.30 ± 26.76	3.28 ± 3.33	3.62 ± 4.25	3.45 ± 3.75
IM	18.42 ± 21.52	18.16 ± 26.04	19.78 ± 25.91	18.79 ± 24.36	3.91 ± 5.76	3.87 ± 5.52	3.89 ± 5.58
VNC	31.16 ± 5.78	31.28 ± 4.21	31.54 ± 4.72	31.33 ± 4.23	8.54 ± 20.45	14.37 ± 32.5	11.55 ± 25.35
FUI	-	-	-	-	70.17 ± 99.10	71.80 ± 99.53	70.98 ± 99.26
	Ø Rater 1/Rater 2				Ø Rater 1/Rater 2		
	ROI 1_R1/2_	ROI 2_R1/2_	ROI 3 _R1/2_	Total mean	Volume 1_R1/2_	Volume 2_R1/2_	Total mean
BW	53.69 ± 25.11	53.15 ± 20.88	55.13 ± 30.00	53.99 ± 25.27	3.07 ± 2.70	3.26 ± 3.23	3.17 ± 2.98
IM	18.34 ± 18.70	18.78 ± 22.74	20.24 ± 27.51	19.12 ± 22.90	3.03 ± 3.47	2.95 ± 3.24	2.99 ± 3.32
VNC	31.37 ± 4.61	30.77 ± 4.19	31.13 ± 4.42	31.09 ± 4.13	5.06 ± 11.26	7.70 ± 16.48	6.44 ± 13.23
FUI	-	-	-	-	60.45 ± 90.44	68.08 ± 100.00	64.27 ± 95.17
	Performance measures from ROC						
	Prediction of infarction						
	Rater 1						
	Cut-off (HU)	Sensitivity (%)	Specificity (%)	LR	True positive (%)	True negative (%)	Accuracy
BW	>48.80	64.71	100.00	*n.a.*	64.71	100.00	0.73
IM	>10.40	94.12	100.00	*n.a.*	94.12	100.00	0.96
VNC	>34.05	94.12	81.82	5.176	94.12	81.82	0.91
	Rater 2						
	Cut-off (HU)	Sensitivity (%)	Specificity (%)	LR	True positive (%)	True negative (%)	Accuracy
BW	>46.64	82.35	90.91	9.059	82.35	90.91	0.84
IM	>10.30	88.24	100.00	*n.a.*	88.24	100.00	0.91
VNC	<31.37	67.65	90.91	7.441	67.65	90.91	0.73
	Ø Rater 1/Rater 2						
	Cut-off (HU)	Sensitivity (%)	Specificity (%)	LR	True positive (%)	True negative (%)	Accuracy
BW	>49.23	76.47	90.91	8.412	76.47	90.91	0.80
IM	>9.97	91.18	100.00	n.a.	91.18	100.00	0.93
VNC	<33.79	94.12	81.82	5.176	94.12	81.82	0.91
	Prediction of haemorrhagic transformation						
	Rater 1						
	Cut-off (HU)	Sensitivity (%)	Specificity (%)	LR	True positive (%)	True negative (%)	Accuracy
BW	>51.12	80.00	71.43	2.800	80.00	71.43	0.73
IM	>17.90	90.00	65.71	2.625	90.00	65.71	0.71
VNC	>28.40	100.00	37.14	1.591	100.00	37.14	0.51
	Rater 2						
	Cut-off (HU)	Sensitivity (%)	Specificity (%)	LR	True positive (%)	True negative (%)	Accuracy
1107	>52.00	80.00	77.14	3.500	80.00	77.14	0.78
1107	>13.19	100.00	57.14	2.333	100.00	57.14	0.67
1107	<33.80	90.00	31.43	1.313	90.00	31.43	0.44
	Ø Rater 1/Rater 2						
	Cut-off (HU)	Sensitivity (%)	Specificity (%)	LR	True positive (%)	True negative (%)	Accuracy
BW	>51.21	80.00	71.43	2.800	80.00	71.43	0.73
IM	>17.13	90.00	62.86	2.423	90.00	62.86	0.69
VNC	<29.13	50.00	68.57	1.591	50.00	68.57	0.64

Results from receiver operator analyses (ROC)

Abbreviations: *BW,* Weighted brain window series; *FUI,* Follow-up imaging; *IM,* Iodine map series; *LR,* Likelihood ratio; *VNC,* Virtual non-contrast series; *ROI,* region of interest; *1-3*, Measurements 1, 2, and 3

**Fig. 2:**
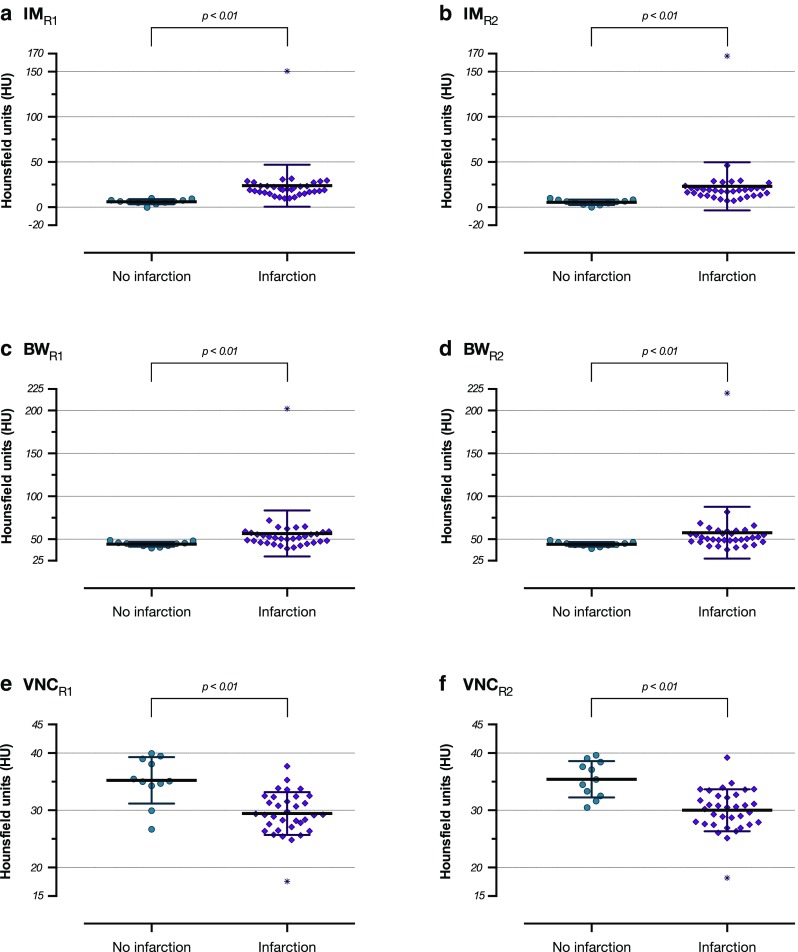
**a**–**f** Scattergrams of the density means ± standard deviations in the groups “no infarction” and “infarction” on the iodine map series for reader 1 (Figure 2a) and reader 2 (Figure 2b), brain window series for reader 1 (Figure 2c) and reader 2 (Figure 2d), and virtual non-contrast series for reader 1 (Figure 2e) and reader 2 (Figure 2f). There are significant differences between the groups (Mann-Whitney tests)

In the IM (Fig. 2a and b) and the BW series (Fig. 2c and d), future non-infarction areas displayed significantly lower densities than future ischemic or haemorrhagic infarction areas and in the VNC series (Fig. 2e and f), future normal brain tissue areas displayed higher densities than ischemic or haemorrhagic infarction areas.

**Fig. 3:**
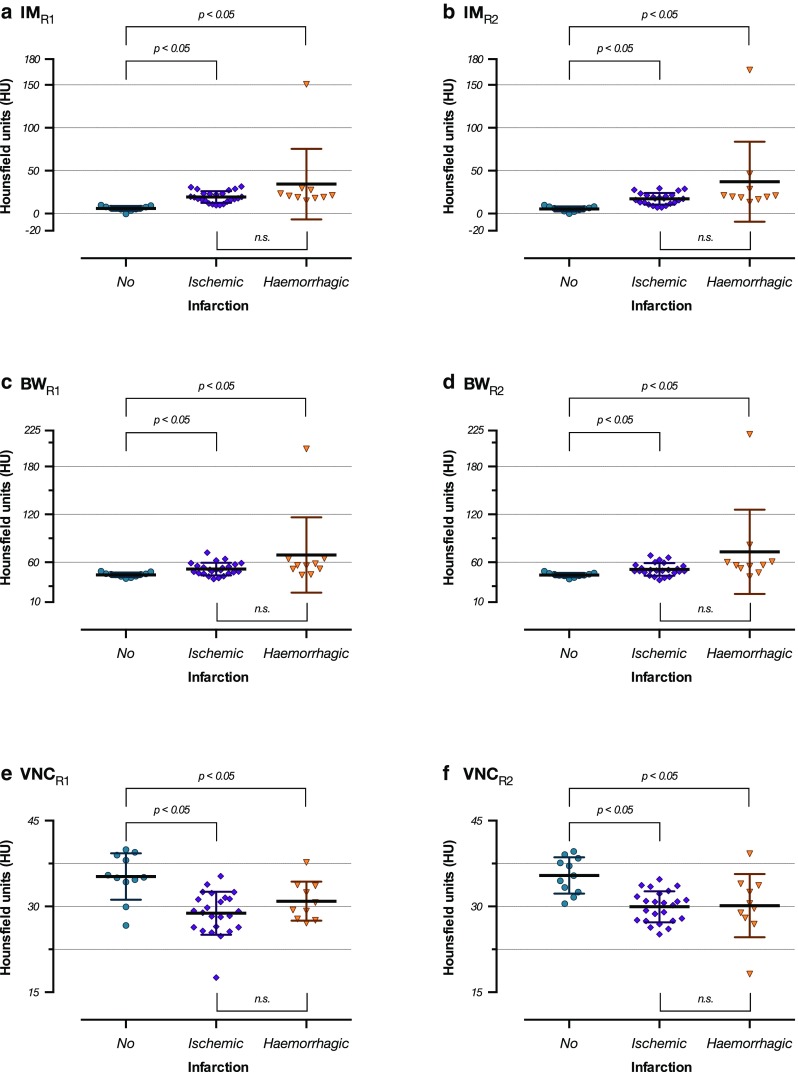
**a**–**f** Scattergrams of the density means ± standard deviations in the groups “no infarction”, “ischemic infarction”, and “haemorrhagic” infarction on the iodine map series for reader 1 (Figure 3a) and reader 2 (Figure 3b), brain window series for reader 1 (Figure 3c) and reader 2 (Figure 3d), and the virtual non-contrast series for reader 1 (Figure 3e) and reader 2 (Figure 3f). There are significant differences between the groups “no infarction” and “ischemic infarction” and “no infarction” and “haemorrhagic infarction”, but not between “ischemic infarction” and “haemorrhagic infarction” (Kruskal-Wallis tests with Dunn’s post hoc tests)

Significantly different densities between the groups “no infarction” and “ischemic infarction” as well as the groups “no infarction” and “haemorrhagic infarction”, but not between the groups “ischemic infarction” and “haemorrhagic infarction” in the IM series (Fig. 3a and b), the BW series (Fig. 3c and d), and the VNC series (Fig. 3e and f) were found.

Neither the quantity of contrast agent injected during the angiography nor any other factor measured correlated significantly with the densities measured in the individual series (Spearman’s ρ ≈ 0.2 each, *p* > 0.05 each). In the patients who had had an MRI as a follow-up examination, the same number of infarctions was found as in the patients who had had a CT follow-up examination (2.1 ± 1.29 versus 2.4 ± 0.97, *p* > 0.05).

**Fig. 4:**
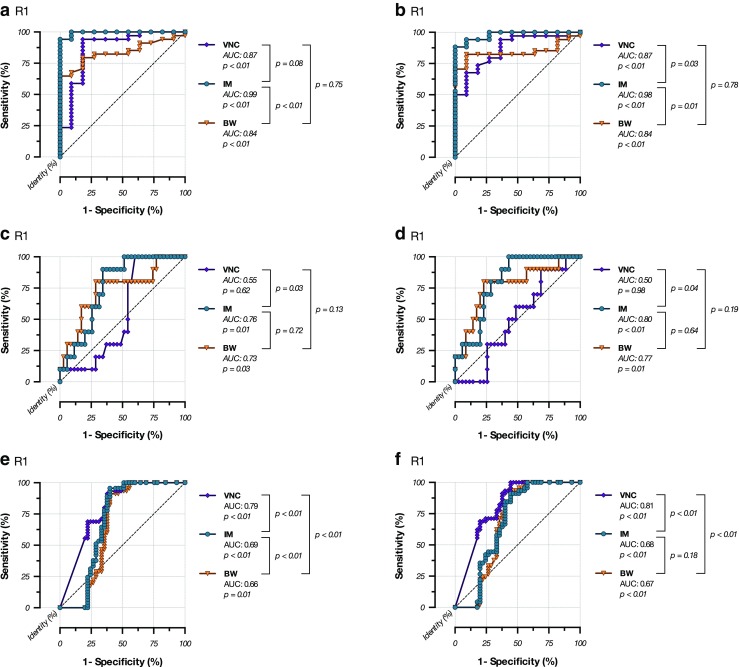
**a**–**f** Receiver operating characteristic analyses. Figure 4a and b; rater 1 and 2: Infarction prediction using densities from IM, VNC, and BW series. The cut-off value for the prediction of an infarction is 10.4 HU (reader 1) and 10.3 HU (reader 2). Figure 4c and d; reader 1 and 2: Haemorrhage prediction using densities from IM, VNC, and BW series. Figure 4e and f; reader 1 and 2: Infarction volume prediction using lesion volumes from IM, VNC, and BW series

The ROC analyses of the IM densities for the differentiation between future infarction areas and non-infarction areas (Fig. 4a and b) revealed an AUC of 0.9947 ± 0.00718 (CI: 0.98061-1.009; *p* < 0.0001) for reader 1 and an AUC of 0.9786 ± 0.01732 (CI: 0.9447-1.013; *p* < 0.0001) for reader 2. The density cut-off value for discrimination was >10.4 HU for reader 1 and >10.3 HU for reader 2. Using these densities, the sensitivity reached 94.12 % for reader 1 and 88.24 % for reader 2, and the specificity was 100 % for both readers. The accuracy of the test was 0.96 for reader 1, and 0.91 for reader 2. The values for the VNC series and the BW series and the summarized values of both raters are presented in Table 3.

The ROC analyses for the differentiation between the future haemorrhagic and non-haemorrhagic infarctions are shown in Fig. 4c and d, the ROC analyses for the prediction of the size of infarctions are shown in Fig. 4e and f, and the measures of performance are shown in Table 3.

## Discussion

In the present cohort of patients with BBB disrupted areas after IAR, approximately one quarter of these areas did not become infarctions, whereas the other areas evolved either to ischemic or haemorrhagic infarctions. Future infarction areas were found to be denser than future non-infarction areas in the BW and IM series and more hypodense in VNC series.

The iodine map allowed extremely good differentiation between future infarction areas and non-infarction areas, with an AUC of 0.99 for rater 1 and an AUC of 0.98 for rater 2. The cut-off value for discrimination was >10.4 HU for reader 1 and >10.3 HU for reader 2, with a sensitivity of 94.12 %/88.24 % and a specificity of 100 %/100 % (Table 3). The BW and the VNC series are of lower value for this purpose. For the prediction of a future haemorrhagic transformation, IM was slightly, but not statistically significantly superior to the BW series with a cut-off of >17.9 HU for rater 1 and >13.19 HU for rater 2 and an AUC of 0.76 for rater 1 and 0.8 for rater 2. Both series are of moderate to good value for this purpose. The volume of an infarction can be predicted moderately to well using the VNC series with an AUC of 0.79 (rater 1) and 0.81 (rater 2). In the IM and BW series, intra- and inter-rater reliability was high, indicating a high reproducibility of this method. The post-processing steps are easy to perform, and the time required is short. Image interpretation can be achieved in less than a minute.

A considerable limitation of the present study is the relatively small number (45) of areas of interest. However, the group differences detected are distinctive and highly significant; therefore, the results appear to be reliable. In some patients early infarct signs, BBB disruptions, or haemorrhage were only poorly demarcated on the post-interventional DECT scan. The poor geographic demarcation due to ill-defined or indistinct margins is reflected in the comparison with the density measurements with low inter- and intra-observer reliability in the volumetry of the lesions. The most relevant limitation might be that the DECT technique is not yet available in all centres performing IAR. Moreover, the technique can be used with different voltages of the second tube. While the first tube was set at a constant 140 kVp, the voltage of the second tube fluctuates depending on the article between 80 [5, 6, 9, 10], 120 [8], and 100 [24] kVp. It is unclear whether this has an effect on the densities measured in the calculated IM or VNC series or the respective reported thresholds and could be a source of error when transferring the technique to another device. This question could be clarified only in a phantom study. Similarly, it is not known whether there would be an effect on the results if instead of the H30 smooth kernel used in this study, a DE34 kernel or an H31 kernel, for example, were used for visualization. Different kernels could not be calculated retrospectively for this study because the raw data required for this are stored for only a few days. Since the kernels are similar, we consider an effect to be unlikely, which was also confirmed by the results thus far of four patients. However, potential effects must be ruled out by a larger number of examinations. Although the quantity of contrast agent injected during the angiography did not correlate significantly with the densities in the individual series, further studies in this respect are certainly necessary. The density of the BBB disrupted areas likely depends primarily on the degree of BBB disruption. However potential effects of other parameters are conceivable. Whether the follow-up imaging was performed using MRI or CT scan did not play a role with respect to the number of the infarctions. One explanation is that the follow-up imaging was performed such a long time (>24 h, <7 days) after IAR that the infarctions were clearly demarcated in the CT scans.

DECT has been described as a reliable tool for distinguishing intracranial hemorrhage from BBB disruptions after intravenous contrast administration [4–6, 8] or neuroendovascular therapy with intraarterial contrast administration [9, 10, 25]. Consistent with the present study, not all BBB disrupted areas represent a later infarction [9], but all infarctions evolved in BBB disrupted areas. However, the present study was the first attempt to investigate the later evolution of BBB disrupted areas by investigating the densities of the different DECT series. Thus, the described method is entirely novel.

In one study in which a conventional CT scan was performed immediately after IAR, a differentiation could be made between BBB disruption and BBB disruption with future haemorrhage using a cut-off value of 50 HU with an AUC of 0.78 [26]. This is very consistent with the results using the assessment of the “brain window” in the present study. Hyperdensity in one area is not necessarily associated with a poor outcome [27], but if the density is greater than 150 HU, a poor outcome is more likely [28]. In fact, this occurred only once in the present study. The IM series is somewhat better in this respect than the “brain window”, while the VNC series has no value for predicting haemorrhage.

Retrospectively, pre-interventional hypodense areas found in the CT scan were early signs of infarction in only two regions. Both were located in very extensive future infarction regions, but were not affected by haemorrhagic transformation. No statistical statement can be made based on these two cases.

In the present study, BBB disruptions after IAR were found in 20 out of 34 cases (58 %), which lies within the known range of between 24 % and 82 % of cases [9, 10, 24, 29]. In addition, patients with BBB disruptions seem to have lower rates of neurologic improvement, higher mortality rates, and a higher risk of haemorrhagic complications [24, 29]. The present study features a smaller population than these studies just mentioned, but it is the first study to focus on the grade of BBB disruption, measured by IM density. In the present study, only areas with BBB disruptions were investigated; therefore, no prognostic estimations from the presence of a BBB disruption in comparison with patients with no detectable BBB disruption can be made.

All BBB disrupted areas were located in the vascular territory dependent on the treated vessel. The prediction of the development of an infarction was less accurate with the BW series than with the VNC and the iodine map series. A hypothetical explanation for this is that all hyperdensities of various causes are visible in the brain window: residual contrast agent in tissue, contrast enhancements, contrast agent extravasation, and haemorrhages. They are mixed with regions that are possibly already hypodense .

A prior study described the limitations of post interventional “conventional” CT concerning the prediction of BBB disruptions evolving to haemorrhagic infarctions [30]. As described in the present study, even with DECT it is possible only to a limited extent to differentiate between a future infarction and a haemorrhagic transformed infarction. The areas under the curve for the BW series, and the IM series were only moderate to good. The frequency of haemorrhagic transformation in this study (22.2 %) was within the range of frequencies of 10 % to 42 % reported in the literature [6, 9, 24, 31], and it can be determined that, consistent with the literature [32], haemorrhagic transformation occurred only in BBB disrupted areas. Early signs of infarction in the pre-interventional CT that present a risk factor for haemorrhagic transformation were found in this study in only two patients, who later developed large infarctions, but no haemorrhage.

Although both the intra-rater and the inter-rater reliability regarding the volume of the lesions was smallest in the VNC series and can be described as weak, the volumes of visible hypodense areas in the VNC maps correlate with the volumes of the ultimate infarctions. The lower VNC densities in future infarction areas found in the present study can most likely be explained as very early infarction demarcations so that they can be considered the precursors of the areas determined in the final follow-up examinations. The volume of the contrast agent applied during the angiography did not correlate with the densities detected, so it can be assumed that the threshold values indicated are independent of the volumes of the quantity of contrast agent within the reported range.

Taking recent developments regarding the combination of reperfusion therapies and neuroprotective measures into consideration [15–17], it seems to be even more important to detect reperfusion injuries as early as possible and to be able not only to predict the development of an infarction, but its volume and the probability of haemorrhage with better reliability. This is all the more important because the patients in the UI [17] study did not have any mechanical thrombectomy, through which greater reperfusion damage is expected than with IV thrombolysis, mainly because it cannot be initiated until later than IV thrombolysis. Additional major efforts will be required to be able detect or even predict reperfusion damage in order to develop an individually adapted treatment strategy for each patient’s condition.

Future infarction transformation of BBB disrupted areas immediately after IAR can be predicted with a high accuracy and a high reproducibility using DECT with IM reconstructions. A higher grade of BBB disruption seems to be connected with future infarction development. The IM is somewhat better suited than the BW series for predicting a future haemorrhagic transformation. However, the predictability of haemorrhagic transformation is only moderate to good, as is the predictability of the infarction volume using the VNC series. This knowledge could help to detect patients at risk for the development of large, malignant infarctions in the subacute stage of a stroke.

## Abbreviations

IAR: Intraarterial recanalisation
BBB: Blood-brain barrier
CT: Computed tomography
MRI: Magnetic resonance imaging
DECT: Dual-energy computed tomography
HU: Hounsfield units
EST: Endovascular stroke therapy
ROC: Receiver operator characteristic
ROI: Region of interest
AUC: Area under the curve
BW: Weighted “brain window” dual-energy series
IM: Iodine map series
VNC: Virtual non-contrast series
SWI: Susceptibility weighed imaging series
